# Prehabilitation for frail patients undergoing total hip or knee replacement: protocol for the Joint PREP feasibility randomised controlled trial

**DOI:** 10.1186/s40814-023-01363-6

**Published:** 2023-08-07

**Authors:** Tanzeela Khalid, Yoav Ben-Shlomo, Wendy Bertram, Lucy Culliford, Clare England, Emily Henderson, Catherine Jameson, Marcus Jepson, Shea Palmer, Michael R. Whitehouse, Vikki Wylde

**Affiliations:** 1 Bristol Medical School, University of Bristol, Bristol, UK; 2 National Institute for Health and Care Research Applied Research Collaboration West at University Hospitals Bristol and Weston NHS Foundation Trust, Bristol, UK; 3 National Institute for Health and Care Research Bristol Biomedical Research Centre, University Hospitals Bristol and Weston NHS Foundation Trust and University of Bristol, Bristol, UK; 4 Centre for Care Excellence, Coventry University and University Hospitals Coventry & Warwickshire NHS Trust, Coventry, UK

**Keywords:** Hip replacement, Knee replacement, Frailty, Prehabilitation, Exercise, Protein, Feasibility study

## Abstract

**Background:**

Approximately, 8% of community-based adults aged ≥ 50 years in England are frail. Frailty has been found to be associated with poorer outcomes after joint replacement. Targeting frailty preoperatively via exercise and protein supplementation has the potential to improve outcomes for people undergoing joint replacement. Prior to proceeding with a randomised controlled trial (RCT), a feasibility study is necessary to address key uncertainties and explore how to optimise trial design and delivery.

**Methods:**

The Joint PRehabilitation with Exercise and Protein (Joint PREP) study is a feasibility study for a multicentre, two-arm, parallel group, pragmatic, RCT to evaluate the clinical and cost-effectiveness of prehabilitation for frail patients undergoing total hip or knee replacement. Sixty people who are ≥ 65 years of age, frail according to the self-reported Groningen Frailty Indicator, and scheduled to undergo total hip or knee replacement at 2–3 hospitals in England and Wales will be recruited and randomly allocated on a 1:1 ratio to the intervention or usual care group. The usual care group will receive the standard care at their hospital. The intervention group will be given a daily protein supplement and will be asked to follow a home-based, tailored daily exercise programme for 12 weeks before their operation, in addition to usual care. Participants will be supported through six follow-up calls from a physiotherapist during the 12-week intervention period. Study questionnaires will be administered at baseline and 12 weeks after randomisation. Embedded qualitative research with patients will explore their experiences of participating, reasons for nonparticipation, and/or reasons for withdrawal or treatment discontinuation. Primary feasibility outcomes will be eligibility and recruitment rates, adherence to the intervention, and acceptability of the trial and the intervention.

**Discussion:**

This study will generate important data regarding the feasibility of a RCT to evaluate a prehabilitation intervention for frail patients undergoing total hip and knee replacement. A future phase-3 RCT will determine if preoperative exercise and protein supplementation improve the recovery of frail patients after primary joint replacement.

**Trial registration:**

ISRCTN11121506, registered 29 September 2022.

**Supplementary Information:**

The online version contains supplementary material available at 10.1186/s40814-023-01363-6.

## Background

Total hip replacement (THR) and total knee replacement (TKR) are two of the most common elective surgical procedures; in the UK, > 200,000 are performed annually in the National Health Service (NHS) [1, 2]. Joint replacement is performed to provide relief from chronic pain and improve functional ability and is successful for many patients [3]. However, there are a proportion of patients who experience adverse post-operative outcomes, including surgical and medical complications, mortality, chronic postsurgical pain, and long-term functional limitations [4–7]. Research has found that although people with frailty often experience improvements in function and pain with joint replacement, they are particularly susceptible to the risks associated with surgery [8–12]. Frailty is a syndrome, whereby decline in physiological reserve and function confers increased vulnerability to physiological stressors such as surgery.

The prevalence of frailty in community-based adults aged ≥ 50 years in England is estimated to be 8% [13]. Studies from North America show that one-third to one-half of those waiting for a joint replacement are frail [10, 14]. In the UK, the average age of those undergoing THR or TKR is approximately 70 years [1], suggesting that many operations are carried out in frail individuals. Following joint replacement, frailty has been associated with increased mortality, length of hospital stay, admission to intensive care, discharge to institutional care, readmissions, and poorer patient-reported outcomes [8–12]. The prevalence of frailty among patients undergoing joint replacement in the NHS is likely to continue to grow due to the ageing population and increased waiting times for surgery [15].

Frailty is potentially modifiable, and studies suggest it may be treated with interventions including exercise and protein supplementation [16–18]. There is a clinical need and opportunity to determine whether targeting frailty in the preoperative phase via protein supplementation and exercise can improve post-operative outcomes in people undergoing joint replacement. The preoperative phase following a patient being placed on the waiting list for surgery provides a window of opportunity to intervene to ensure that patients undergo operations in an optimised physiological state. However, prior to proceeding with a randomised controlled trial (RCT), a feasibility study is necessary to address key uncertainties regarding whether a RCT is possible and explore how to optimise trial design and delivery.

## Methods

This protocol follows guidance from Standard Protocol Items: Recommendations for Interventional Trials (SPIRIT) [19]. A SPIRIT schedule of enrolment, interventions, and assessment is provided in Table 1, and a SPIRIT checklist is provided in Additional file 1.

**Table 1:**
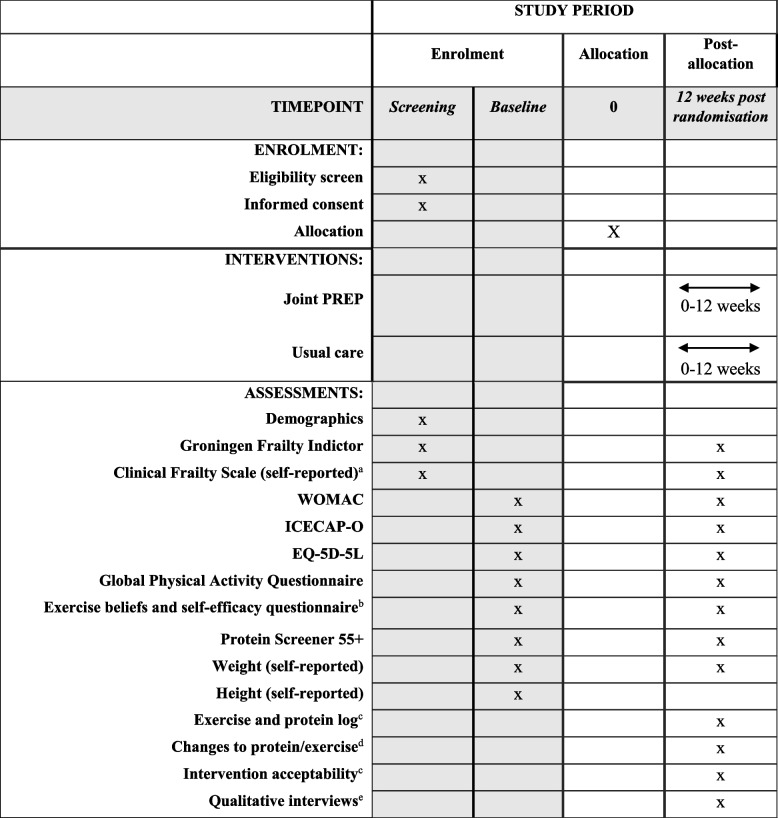
SPIRIT schedule of enrolment, interventions, and assessments

^a^Clinical Frailty Scale will be completed by a clinician when the patient attends the hospital for their routine preoperative assessment appointment

^b^Self-efficacy subscale only at 12-week post-randomisation

^c^Intervention group only

^d^Usual care group only

^e^Subsample only

### Aims and objectives

The aim of the study is to estimate the extent to which a full-scale RCT of the clinical and cost-effectiveness of preoperative protein supplementation and structured exercise intervention in frail individuals waiting for a THR or TKR is feasible. Specific objectives are to estimate eligibility, recruitment and retention rates, adherence to and acceptability of the trial, data completion rates, and efficacy potential.

### Design

Joint PRehabilitation with Exercise and Protein (Joint PREP) is a feasibility study for a multicentre, two-arm, parallel group, pragmatic, RCT. The study will be conducted at 2 to 3 NHS elective orthopaedic centres. Embedded qualitative research with patients will explore their motivation to take part and experiences of participating, reasons for nonparticipation, and/or reasons for withdrawal or treatment discontinuation. Group discussions with site staff will elicit views on organisational barriers or facilitators for the RCT and explore positions of equipoise and acceptability of the study. The reporting of this protocol follows the reporting guideline for pilot and feasibility studies [20].

### Regulatory approvals

Ethics approval was obtained from the East of Scotland NHS Research Ethics Committee 2 on 30th August 2022 (reference 22/ES/0033) and Health Research Authority (HRA) approval on 6th September 2022. The study is registered on the International Standard Randomised Controlled Trial Number registry (ISRCTN, reference: ISRCTN11121506). Any protocol amendments will be submitted to the HRA for approval prior to implementation and updated on the ISRCTN registry. All patients will provide informed, written consent prior to study participation.

### Patient and public involvement in study design

This study was designed in collaboration with a patient and public involvement (PPI) group, called the Patient Experience Partnership in Research Group. The group consists of patients with experience of osteoarthritis and joint replacement. Through group meetings, patient representatives have worked with the research team to codesign the intervention and study, including testing protein supplements, providing feedback on the exercise component and documents, preparing patient-facing study documents, and developing the patient interview topic guide. The research team and PPI group will continue to work together during the study to interpret study findings, develop strategies and outputs for public dissemination, and plan a grant application for a RCT if this study demonstrates that a trial is feasible.

### Patient recruitment

**Fig. 1:**
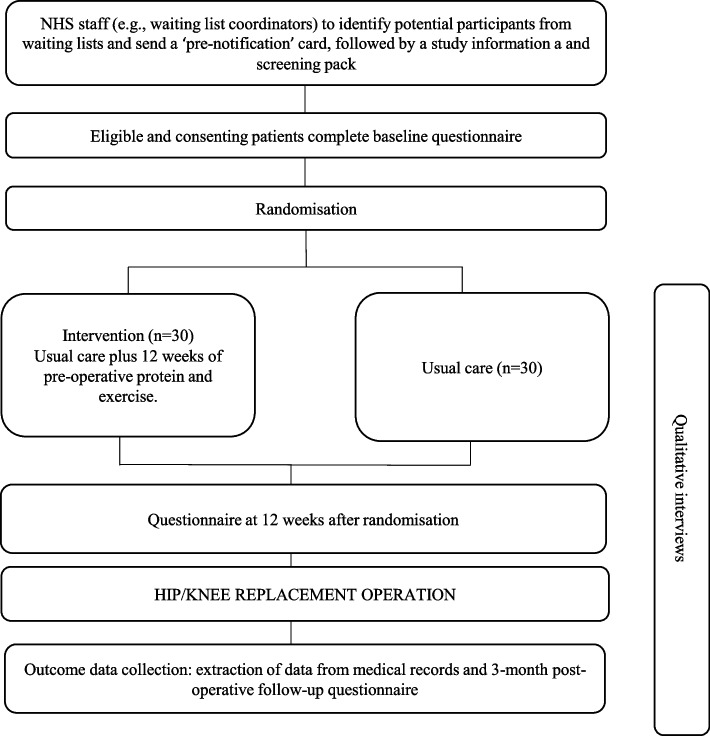
Participant flow

An overview of participant flow is provided in Fig. 1.

#### Eligibility criteria

Inclusion criteria are patients scheduled for primary THR or TKR, ≥ 12 weeks until intended date of operation, ≥ 65 years of age, and frail according to self-reported Groningen Frailty Indicator (score of ≥ 4) (29). Exclusion criteria include contraindications to following study treatments (e.g. following a low-protein diet or comorbidities which preclude participation in exercise), participating in another study that may affect the outcomes of this feasibility study or that does not permit co-enrollment in another study or where co-enrollment would be burdensome to the patient, or unable or unwilling to provide informed consent.

#### Screening and recruitment

NHS patients on waiting lists for a THR or TKR who are aged ≥ 65 years and within an appropriate window prior to their anticipated date of surgery (i.e. as close to 12 weeks prior to surgery as possible) will be identified by the clinical care team and sent a study information pack and screening questionnaire. The screening questionnaire includes demographic questions, the Groningen Frailty Indicator [21], the self-reported version of the Clinical Frailty Scale [22], and questions about ability to participate in exercise, consume extra protein, and participate in other studies. Interested patients are asked to complete and return the screening questionnaire and consent form in a pre-paid self-addressed envelope. One reminder pack will be sent if no response is received within 1 to 2 weeks, followed by a telephone call to ask if they would like any additional support to participate, such as completing the questionnaire over the telephone. Patients who return a screening questionnaire and consent form and meet the eligibility criteria will then be telephoned by the local research team to confirm that they fully understand what participation involves, answer any questions, and explain what will happen during the study. Participants will then be sent a baseline questionnaire to complete. All patients meeting the eligibility criteria will be invited to consider participation in a telephone/online semi-structured interview.

### Randomisation

Participants will be randomised by a member of the local research team using a computer-based tool (REDCap), stratified by study site and operation type (THR/TKR), with full allocation concealment. Randomisation will be conducted as soon as possible after the research team receive a completed baseline questionnaire. Participants will be informed of their treatment allocation by letter, followed by a telephone call for participants randomised to the intervention group to discuss arrangements for the intervention. Blinding of study participants will not be possible due to the nature of this intervention. After participants have been randomised, a letter will be sent to their GP to inform them of their participation in the Joint PREP study and their treatment allocation.

### Usual care

All participants in the study will receive their usual care. Participants randomised to the usual care group will not receive any additional advice or treatment to that provided routinely by their healthcare team. Usual care can vary between NHS trusts but may involve education classes, physiotherapy, group exercise, occupational therapy, and surgeon review before and after joint replacement. We will capture what constitutes usual care at each study site and will record details of any other preoperative exercise or nutrition programme(s) the participant is following as part of their usual care.

### Intervention

Participants randomised to the intervention arm will undertake 12 weeks of a preoperative exercise and protein supplementation intervention. The intervention was designed as a personalised and home-based programme with regular contact and support to maximise success [23]. An intervention duration of 12 weeks was informed by previous research that at least this duration of exercise is required to have benefits relevant to frailty [18]. Reporting of the intervention follows guidance from the template for intervention description and replication (TIDieR) [24], and a TIDieR checklist is provided in Additional file 2.

#### Exercise

Participants will have an initial 1:1 appointment with a trained physiotherapist (either in person at the hospital or virtually via a secure video platform) to introduce the intervention and develop a 12-week individualised home-based exercise programme. A risk assessment will be carried out and will inform adaptation of exercises and progression schedule to ensure they are tailored to individual physical capabilities and to minimise risk. Appropriate progressions (difficulty and number of repetitions) will be agreed, and participants will be supported with telephone calls every 2 weeks to gradually increase their levels of moderate physical activity throughout the intervention period. The exercises and resources are based on the NEMEX-TJR programme (https://nemex.trekeducation.org) and have been adapted for home use with permission from the Translating Research Evidence and Knowledge programme at La Trobe University, Australia (https://trekeducation.org). The exercises include a warmup, pelvic lifts, sit-ups, lunges, sideway lunges, knee flexion and extension, hip abduction and adduction, chair stands, step-ups, and a cool down. Participants will be provided with two booklets to assist them with completing the exercises. One booklet will have instructions and illustrations for each of the exercises, and the other will have more general information about maintaining exercise, goal setting, pacing, and how best to deal with setbacks. Participants will also be offered the option of signing up to an online platform to support the home-exercise programme. All participants will be issued with Therabands (of varying resistance) for the knee and hip exercises, and where appropriate, participants will be issued with an exercise step and/or gym ball.

#### Protein

Participants will be asked to consume 20 g of additional protein in the form of one pot of jelly (118 ml) each day for 12 weeks prior to their surgery (ProSource jelly from NUTRINOVO, Wiltshire, UK). Participants who do not eat gelatine will be offered an alternative product vanilla-flavoured whey or fava bean protein powder or chocolate pea protein powder (Pulsin, Gloucester, UK) that does not contain gelatine. Participants will be asked to consume the protein within 3 h after exercise as muscle protein synthesis peaks during this time [25]. They will also be advised to consume the protein between meals to minimise any effect on appetite. Participants will be provided with instructions for eating the jelly pots or making up and drinking the powder shakes. Batches of jelly pots/protein powder will be posted to participants at regular intervals to avoid the need to store large quantities in their home. Provision of protein supplements will cease after 12 weeks; however, participants will have the option to continuing to purchase their own protein supplements after this time period.

#### Telephone follow-up calls

Participants will be telephoned by a physiotherapist six times over the 12-week intervention period, at approximately 1, 2, 4, 6, 8, and 10 weeks. The purpose of the telephone calls will be to check that participants are coping with the exercises and the additional protein, review and consider appropriate progression of exercises, and to address any problems or concerns participants may have. In the final telephone call, continuation of the home exercise programme until the time of surgery (if relevant) will be discussed.

#### Intervention training

Physiotherapists delivering the intervention will have attended a half-day training session from the study team nutritionist and physiotherapist and will have been provided with an intervention training manual. They will have experience of working clinically with patients undergoing joint replacement.

#### Assessment of contamination

An estimate of protein adequacy in usual diet and usual physical activity data will be collected from all participants via study questionnaires to explore if participation in the study has influenced the behaviour of participants randomised to the usual care group. Participants in the usual care group will also be asked if they made any changes to their diet or physical activity over the 12-week intervention period.

### Questionnaires and data collection

All participants will be asked to complete questionnaires at baseline (after recruitment and prior to randomisation) and 12 weeks after randomisation. Questionnaires will be administered on paper or online, depending on participant preference. Nonresponders will be followed up with a reminder questionnaire and then a telephone call from the local research team to offer the option of completing the outcome measures over the telephone.

A schedule of assessment is provided in Table 1. Pain and function will be assessed using the Western Ontario and McMaster Universities Osteoarthritis Index (WOMAC) [26]. Health-related quality of life will be measured using the EQ-5D-5L [27] and capabilities with the ICECAP-O [28]. Other measures include the Groningen Frailty Indicator, Clinical Frailty Scale, Global Physical Activity Questionnaire [29], Exercise Self-Efficacy and Beliefs Questionnaire [30], and Protein Screener 55 + [31]. Participants will be asked to self-report their height and weight to allow BMI to be calculated. Participants in the usual care group will be asked about any changes to usual diet or usual exercise/physical activity in the past 12 weeks and, if so, what these changes were. Participants in the intervention group will be asked questions on the acceptability of the intervention.

The Clinical Frailty Scale will be completed by a healthcare professional when the patient attends the hospital for their routine preoperative assessment appointment so that self-reported and clinician-assessed versions of the Clinical Frailty Scale can be compared. Data will be extracted from participants’ medical records on preoperative comorbidities, indication for surgery, surgery details, length of hospital stay, discharge destination, preadmission residence, post-operative mobilisation, and post-operative complications up to 30 days after surgery (if surgery takes place within the study timeframe).

### Outcome assessment and progression criteria

#### Primary feasibility outcomes

The primary outcome is to determine the feasibility of a definitive RCT. This will involve evaluation of the key parameters of uncertainty for a RCT which are eligibility and recruitment rates, adherence to the intervention, and acceptability of the trial and the intervention. Eligibility and recruitment will be assessed by collecting detailed screening and recruitment records to allow review of the number of eligible, approached, and consented patients, alongside information on reasons for non-eligibility and nonparticipation. To assess adherence to the intervention, participants will be provided with a log form and asked to keep a daily record of whether they eat their protein and complete their exercises. Further details about reasons for non-adherence will be recorded on a proforma during the intervention telephone calls. Participants will be considered to have adhered to the intervention if they consumed the protein on at least 4 days per week for at least 10 weeks and/or completed the exercises at least 3 days per week for at least 10 weeks (or 80% of intervention duration if the time available for the intervention is shorter than 12 weeks, e.g. if the participant is offered an operation date earlier than expected). Acceptability of the trial and intervention will be evaluated through qualitative interviews, questions in the study questionnaires, retention rates, and reasons for withdrawal.

#### Secondary feasibility outcomes

Completion rates for questionnaires administered at baseline and 12 weeks after randomisation will be calculated. We will estimate the efficacy potential of the intervention using the preoperative WOMAC score at 12 weeks after randomisation. The primary outcome for a future full RCT would be in the post-operative period to reflect that prehabilitation aims to optimise post-operative outcomes. While we are not collecting post-operative patient-reported outcomes in this feasibility study due to our study timelines, preoperative pain and function will give an indication of efficacy potential as they are the strongest predictors of post-operative function and pain after joint replacement [32, 33].

#### Progression criteria for a full-scale RCT

The key feasibility issues will be considered on a red (stop), amber (amend), and green (proceed) traffic light system. The feasibility of a full trial will be determined by overall recruitment, retention, and adherence rates. Recruitment will be considered achievable if the rate observed is > 23% (lower limit of the 95% confidence interval for a 30% recruitment rate based on 200 screened). Similar criteria will be applied for retention: i.e. > 70%, for an 80% retention rate based on 60 participants, for adherence: > 37%, and for a 50% adherence rate based on 60 participants. If all three elements fail to reach the threshold (i.e. recruitment is ≤ 23%, retention is ≤ 69%, and adherence is ≤ 37%), a RCT will not be considered feasible. If one or two elements fail to reach the threshold, we will consider how the trial can be modified to address the shortfall. Examples may include modifying recruitment processes or study documents (recruitment), providing additional support/advice (retention and adherence), or offering alternative protein-rich products/exercises (retention and adherence). If all three elements are above the threshold, the trial will be considered feasible.

### Safety

Data on adverse events and serious adverse events will be collected and monitored by the research team to ensure the ongoing safety of participants. All serious adverse events during the intervention period will be notified and reviewed by the study sponsor (North Bristol NHS Trust).

### Withdrawal

Participants may choose to withdraw from the study at any time. Participants who withdraw will be invited to provide their reasons if they wish to do so, and these will be recorded on a proforma to allow identification of barriers to participating and highlight measures to facilitate continued participation in a future RCT.

### Embedded qualitative study

#### Patient interviews

Patients who are approached for the study will be invited to take part in a semi-structured face-to-face/telephone/online interview (according to preference) with a trained qualitative researcher. Interviews with patients who agree to be randomised in the feasibility study will explore their experiences of participating. For those who decline to be randomised or withdraw, interviews will explore reasons for nonparticipation and/or reasons for withdrawal or treatment discontinuation. Target numbers are around 15 to 20 patients who agree to be randomised, and 10 to 15 patients who decline to be randomised and/or are randomised but subsequently withdraw/discontinue treatment, with precise numbers of interviews being determined by principles of data saturation. Interviews will follow a topic guide, which covers experiences of randomisation, tolerability of the intervention, experience of participation, and data collection methods and any barriers or enabling factors that participants experienced in adhering to the intervention. For patients who decline to participate in the feasibility study, the questions will focus on reasons for declining or withdrawing and any barriers to participation.

#### Local research delivery team discussion groups

Group discussions with members of the local research delivery team from each site will be held to elicit their acceptability of the study design, their preference, and margins of equipoise and to understand any potential organisational barriers to RCT implementation and potential mitigation strategies. These will take place once the site has been open to recruitment for approximately 3–6 months to allow for reflection on the experiences of undertaking the study. The discussion groups will take place in person, at local sites, or online (guided by the preferences of the participants) and will be facilitated by the qualitative team. The group discussions will be recorded for the purposes of minute taking only, and the recording will then be deleted once the minutes have been written up. We will not seek consent as the purpose of the discussion is to inform the design of a future RCT rather than generate research data.

### Sample size

As this is a feasibility study, we have based our sample size on recruitment rate. If we identify 200 eligible patients, we can estimate a recruitment rate of 30% (i.e. 60 participants) to within a 95% confidence interval of ± 6.35%.

### Data management

Study data will be stored in the REDCap secure online data capture system. Data validation will be completed in REDCap and any data queries resolved with the site trial team. Participants’ personal data will be stored securely and will only be accessible to trial staff and authorised personnel. All study documents will be made available on request for monitoring and audit by the sponsor or the research ethics committee.

### Statistical analysis

This study is not powered to look at differences in outcomes between the intervention and usual care group. Data on recruitment, retention, and adherence will be reported using frequencies and proportions with 95% confidence intervals. Sample characteristics and outcome data will be summarised by means and standard deviations, medians and interquartile ranges, or frequencies and proportions as appropriate. Preoperative WOMAC scores will be summarised by group and compared descriptively to estimate the efficacy potential of the intervention.

### Qualitative data analysis

Audio recordings of interviews will be transcribed and anonymised. Data will be analysed using thematic analysis, guided by the constant comparison method utilised in the Qualitative Research Integrated within Trials (QuinteT) Recruitment Intervention [34]. A coding index, based on the interview topic guide, will be used to sort the data into themes. An inductive approach to analysis will be used, allowing emergent themes to alter the coding as the analysis progresses. Coding will be completed by a single researcher and then reviewed by a second researcher to ensure both consistency of coding and grounding in the original data. Any inconsistencies in themes or coding will be discussed and resolved between the two researchers. This process will take place in parallel with the data collection to allow any emerging themes to be further explored in subsequent interviews. Qualitative data analysis will be assisted by NVivo software.

### Dissemination plans

We will develop a comprehensive plan for dissemination in collaboration with our PPI group. We anticipate that findings will be disseminated via plain language summaries, conference presentations, and publications in peer-reviewed journals. The main output from this study is data on whether a future RCT is feasible.

### Study status

Recruitment for the study began in December 2022, and the study is anticipated to be complete by December 2023.

## Discussion

To our knowledge, this is the first study to assess the feasibility of a RCT to evaluate the clinical and cost-effectiveness of preoperative exercise and protein supplementation for frail patients undergoing primary TKR or THR in a high-income country setting. By undertaking feasibility work to address key uncertainties, this study will generate important data on the likely success of a future RCT and provide insight into approaches to optimise trial design and processes. A RCT evaluating a prehabilitation intervention for frail patients undergoing joint replacement would provide evidence to guide decisions by patients, clinicians, and policymakers and inform service provision. If shown to be clinically and cost-effective, the Joint PREP intervention could optimise health before surgery and improve outcomes for frail patients undergoing joint replacement.

Systematic reviews of exercise/physiotherapy prior to joint replacement have generally found evidence of limited benefit to post-operative outcomes such as pain, function, quality of life, length of stay, or costs [35, 36]. However, few trials have been conducted specifically to evaluate exercise interventions for frail individuals prior to surgery. Two pilot RCTs evaluated the feasibility of 3–6 weeks preoperative physiotherapy for frail patients [37, 38]. Neither study saw effects on outcomes such as length of stay (although they were not powered to detect this), but recruitment rates were considerably higher for the home-based (70%) than gym-based (34%) intervention. A published protocol for a feasibility RCT in Canada is investigating a 3- to 10-month multicomponent prehabilitation intervention in frail individuals undergoing THR or TKR [39], with the intervention comprising community-based or home-based exercise, protein supplementation, vitamin D supplementation, and medication review. Further studies evaluating prehabilitation interventions are needed to generate a robust evidence base to inform healthcare services.

Protein supplementation is an important component of prehabilitation. Large observational studies have shown inverse associations between protein intakes and risk of frailty [40, 41]. It has been suggested that exercise sensitises muscles to dietary protein, resulting in more of the available amino acids being synthesised into skeletal muscle protein [42]. A systematic review of studies in older people showed the combination of protein and muscle-strengthening exercise was associated with improvements in lean mass, leg strength, and walking capability [43]. International guidelines also suggest the combination of exercise and protein may improve frailty, gait speed, grip strength, and physical performance in older adults with frailty/prefrailty [44], and the European Society for Clinical Nutrition and Metabolism recommends that healthy older people should consume at least 1.0–1.2 g protein/kg body weight/day and take part in daily exercise [45].

In conclusion, this feasibility study is being conducted in preparation for a RCT to generate high-quality evidence on a prehabilitation intervention which aims to improve outcomes for frail patients undergoing THR or TKR. Research using linked NHS primary and secondary care electronic health records demonstrated that most patients with frailty do experience improvement in symptoms with joint replacement, although frailty is associated with worse pain and function after surgery [12]. Research is needed to understand how to optimise the amount of benefit that frail patients experience when undergoing TKR or THR.

### Supplementary Information

**Additional file 1. **SPIRIT 2013 Checklist.

**Additional file 2. **TIDieR (Template for Intervention Description and Replication) Checklist.

## Data Availability

The data sets generated from this study will be available in the University of Bristol Research Data Repository. Data will be available within 6 months following publication of the feasibility study findings. Access to the data will be restricted to ensure that data are only made available to bona fide researchers for ethically approved research projects, on the understanding that confidentiality will be maintained, and after a data access, agreement has been signed by an institutional signatory.

## Abbreviations

HRA: Health Research Authority
ISRCTN: International Standard Randomised Controlled Trial Number
Joint PREP: Joint PRehabilitation with Exercise and Protein
NHS: National Health Service
PPI: Patient and public involvement
QuinteT: Qualitative Research Integrated within Trials
SPIRIT: Standard Protocol Items: Recommendations for Interventional Trials
THR: Total hip replacement
TIDieR: Template for intervention description and replication
TKR: Total knee replacement
WOMAC: Western Ontario and McMaster Universities Osteoarthritis Index
