# Supplementary material for: Prehabilitation for frail patients undergoing total hip or knee replacement: protocol for the Joint PREP feasibility randomised controlled trial
Source: Pilot Feasibility Stud. 2023 Aug 7;9:138. doi: 10.1186/s40814-023-01363-6 (PMC10405490; doi:10.1186/s40814-023-01363-6)
Supplement: Supplementary file 1 — Additional file 1. SPIRIT 2013 Checklist. [file 40814_2023_1363_MOESM1_ESM.doc]

**Additional file 1: SPIRIT 2013 Checklist**

| Section/item | Item No | Description | Addressed on page number |
| --- | --- | --- | --- |
| **Administrative information** | | |  |
| Title | 1 | Descriptive title identifying the study design, population, interventions, and, if applicable, trial acronym | Title |
| Trial registration | 2a | Trial identifier and registry name. If not yet registered, name of intended registry | Abstract |
|  | 2b | All items from the World Health Organization Trial Registration Data Set | Provided after table |
| Protocol version | 3 | Date and version identifier | Version 3, 24-11-22. Amendments to protocol will be reported on ISRCTN record |
| Funding | 4 | Sources and types of financial, material, and other support | Funding |
| Roles and responsibilities | 5a | Names, affiliations, and roles of protocol contributors | Authors’ contributions |
|  | 5b | Name and contact information for the trial sponsor | WHO Trial Registration Data set |
|  | 5c | Role of study sponsor and funders, if any, in study design; collection, management, analysis, and interpretation of data; writing of the report; and the decision to submit the report for publication, including whether they will have ultimate authority over any of these activities | Funding |
|  | 5d | Composition, roles, and responsibilities of the coordinating centre, steering committee, endpoint adjudication committee, data management team, and other individuals or groups overseeing the trial, if applicable (see Item 21a for data monitoring committee) | N/A – no steering committee |
| Introduction |  |  |  |
| Background and rationale | 6a | Description of research question and justification for undertaking the trial, including summary of relevant studies (published and unpublished) examining benefits and harms for each intervention | Background |
|  | 6b | Explanation for choice of comparators | Background |
| Objectives | 7 | Specific objectives or hypotheses | Aims and objectives |
| Trial design | 8 | Description of trial design including type of trial (eg, parallel group, crossover, factorial, single group), allocation ratio, and framework (eg, superiority, equivalence, noninferiority, exploratory) | Methods - Design |
| Methods: Participants, interventions, and outcomes | | |  |
| Study setting | 9 | Description of study settings (eg, community clinic, academic hospital) and list of countries where data will be collected. Reference to where list of study sites can be obtained | Methods - Design, ISRTCN record |
| Eligibility criteria | 10 | Inclusion and exclusion criteria for participants. If applicable, eligibility criteria for study centres and individuals who will perform the interventions (eg, surgeons, psychotherapists) | Methods - Patient recruitment |
| Interventions | 11a | Interventions for each group with sufficient detail to allow replication, including how and when they will be administered | Methods - intervention |
|  | 11b | Criteria for discontinuing or modifying allocated interventions for a given trial participant (eg, drug dose change in response to harms, participant request, or improving/worsening disease) | Methods - intervention |
|  | 11c | Strategies to improve adherence to intervention protocols, and any procedures for monitoring adherence (eg, drug tablet return, laboratory tests) | Methods - intervention |
|  | 11d | Relevant concomitant care and interventions that are permitted or prohibited during the trial | Methods – usual care |
| Outcomes | 12 | Primary, secondary, and other outcomes, including the specific measurement variable (eg, systolic blood pressure), analysis metric (eg, change from baseline, final value, time to event), method of aggregation (eg, median, proportion), and time point for each outcome. Explanation of the clinical relevance of chosen efficacy and harm outcomes is strongly recommended | Methods - outcome measurement |
| Participant timeline | 13 | Time schedule of enrolment, interventions (including any run-ins and washouts), assessments, and visits for participants. A schematic diagram is highly recommended (see Figure) | Figure 1 |
| Sample size | 14 | Estimated number of participants needed to achieve study objectives and how it was determined, including clinical and statistical assumptions supporting any sample size calculations | Methods – sample size |
| Recruitment | 15 | Strategies for achieving adequate participant enrolment to reach target sample size | Methods – patient recruitment |
| **Methods: Assignment of interventions (for controlled trials)** | | |  |
| Allocation: |  |  |  |
| Sequence generation | 16a | Method of generating the allocation sequence (eg, computer-generated random numbers), and list of any factors for stratification. To reduce predictability of a random sequence, details of any planned restriction (eg, blocking) should be provided in a separate document that is unavailable to those who enrol participants or assign interventions | Methods - randomisation |
| Allocation concealment mechanism | 16b | Mechanism of implementing the allocation sequence (eg, central telephone; sequentially numbered, opaque, sealed envelopes), describing any steps to conceal the sequence until interventions are assigned | Methods - randomisation |
| Implementation | 16c | Who will generate the allocation sequence, who will enrol participants, and who will assign participants to interventions | Methods - randomisation |
| Blinding (masking) | 17a | Who will be blinded after assignment to interventions (eg, trial participants, care providers, outcome assessors, data analysts), and how | Methods - randomisation |
|  | 17b | If blinded, circumstances under which unblinding is permissible, and procedure for revealing a participant’s allocated intervention during the trial | N/A |
| **Methods: Data collection, management, and analysis** | | |  |
| Data collection methods | 18a | Plans for assessment and collection of outcome, baseline, and other trial data, including any related processes to promote data quality (eg, duplicate measurements, training of assessors) and a description of study instruments (eg, questionnaires, laboratory tests) along with their reliability and validity, if known. Reference to where data collection forms can be found, if not in the protocol | Methods – outcome measurement |
|  | 18b | Plans to promote participant retention and complete follow-up, including list of any outcome data to be collected for participants who discontinue or deviate from intervention protocols | Methods - withdrawal |
| Data management | 19 | Plans for data entry, coding, security, and storage, including any related processes to promote data quality (eg, double data entry; range checks for data values). Reference to where details of data management procedures can be found, if not in the protocol | Methods – data management |
| Statistical methods | 20a | Statistical methods for analysing primary and secondary outcomes. Reference to where other details of the statistical analysis plan can be found, if not in the protocol | Methods – statistical analysis |
|  | 20b | Methods for any additional analyses (eg, subgroup and adjusted analyses) | Methods – statistical analysis and qualitative analysis |
|  | 20c | Definition of analysis population relating to protocol non-adherence (eg, as randomised analysis), and any statistical methods to handle missing data (eg, multiple imputation) | Methods – intervention and statistical analysis |
| **Methods: Monitoring** | | |  |
| Data monitoring | 21a | Composition of data monitoring committee (DMC); summary of its role and reporting structure; statement of whether it is independent from the sponsor and competing interests; and reference to where further details about its charter can be found, if not in the protocol. Alternatively, an explanation of why a DMC is not needed | N/A |
|  | 21b | Description of any interim analyses and stopping guidelines, including who will have access to these interim results and make the final decision to terminate the trial | N/A |
| Harms | 22 | Plans for collecting, assessing, reporting, and managing solicited and spontaneously reported adverse events and other unintended effects of trial interventions or trial conduct | Methods - safety |
| Auditing | 23 | Frequency and procedures for auditing trial conduct, if any, and whether the process will be independent from investigators and the sponsor | Methods – data management |
| Ethics and dissemination | | |  |
| Research ethics approval | 24 | Plans for seeking research ethics committee/institutional review board (REC/IRB) approval | Methods – regulatory approvals |
| Protocol amendments | 25 | Plans for communicating important protocol modifications (eg, changes to eligibility criteria, outcomes, analyses) to relevant parties (eg, investigators, REC/IRBs, trial participants, trial registries, journals, regulators) | Methods – regulatory approvals |
| Consent or assent | 26a | Who will obtain informed consent or assent from potential trial participants or authorised surrogates, and how (see Item 32) | Methods – patient recruitment |
|  | 26b | Additional consent provisions for collection and use of participant data and biological specimens in ancillary studies, if applicable | N/A |
| Confidentiality | 27 | How personal information about potential and enrolled participants will be collected, shared, and maintained in order to protect confidentiality before, during, and after the trial | Methods – data management |
| Declaration of interests | 28 | Financial and other competing interests for principal investigators for the overall trial and each study site | Competing interests |
| Access to data | 29 | Statement of who will have access to the final trial dataset, and disclosure of contractual agreements that limit such access for investigators | Availability of data and materials |
| Ancillary and post-trial care | 30 | Provisions, if any, for ancillary and post-trial care, and for compensation to those who suffer harm from trial participation | N/A |
| Dissemination policy | 31a | Plans for investigators and sponsor to communicate trial results to participants, healthcare professionals, the public, and other relevant groups (eg, via publication, reporting in results databases, or other data sharing arrangements), including any publication restrictions | Methods – dissemination plan |
|  | 31b | Authorship eligibility guidelines and any intended use of professional writers | Authors’ contributions |
|  | 31c | Plans, if any, for granting public access to the full protocol, participant-level dataset, and statistical code | Availability of data and materials |
| Appendices |  |  |  |
| Informed consent materials | 32 | Model consent form and other related documentation given to participants and authorised surrogates | Available on request |
| Biological specimens | 33 | Plans for collection, laboratory evaluation, and storage of biological specimens for genetic or molecular analysis in the current trial and for future use in ancillary studies, if applicable | N/A |

**World Health Organization Trial Registration Data Set**

| Item number | Item | Description |
| --- | --- | --- |
| 1 | Primary registry and trial identifying number | ISRCTN11121506 |
| 2 | Date of registration in  primary registry | 29/09/2022 |
| 3 | Secondary identifying  numbers | REC reference: 22/ES/0033  IRAS number: 312883  NIHR Research for Patient Benefit reference: NIHR202289 |
| 4 | Sources of monetary or material support | NIHR Research for Patient Benefit reference |
| 5 | Primary sponsor | North Bristol NHS Trust  Research and Innovation, Learning and Research Building, Southmead Hospital, Bristol, BS10 5NB  Tel: 0117 414 9330  E-mail: research@nbt.nhs.uk |
| 6 | Secondary sponsor | Not applicable |
| 7 | Contact for public queries | Tanzeela Khalid  Musculoskeletal Research Unit, Bristol Medical School, University of Bristol  Learning & Research Building, Southmead Hospital, Bristol, BS10 5NB  +44 117 455 1561  t.khalid@bristol.ac.uk |
| 8 | Contact for scientific queries | As above |
| 9 | Public title | Joint PREP: Joint PRehabilitation with Exercise and Protein |
| 10 | Scientific title | A randomised controlled feasibility trial of a prehabilitation intervention in frail older people undergoing total hip or knee replacement |
| 11 | Countries of recruitment | UK |
| 12 | Health condition(s) or problem(s) studied | Total hip and knee replacement surgery |
| 13 | Intervention(s) | Intervention group: Participants will be given a daily protein supplement to add to their diet and will be asked to follow a home-based, tailored daily exercise programme for up to 12-weeks before their operation. All exercises will be demonstrated by a physiotherapist either in-person at a hospital appointment, online or using a hybrid model. Appropriate starting levels will be based on individual physical capacity and risk assessment, and participants will be supported to gradually increase their levels of moderate physical activity throughout the weeks leading up to their surgery through regular telephone/videocalls from a physiotherapist.  Usual care group: Participants will receive usual care for the duration of the study. |
| 14 | Key inclusion and exclusion criteria | Inclusion criteria  1. Scheduled for elective primary total hip or knee replacement  2. >=12 weeks until intended date of operation  3. >=65 years of age  4. Frail according to self-report Groningen Frailty Indicator (score of >4)  Exclusion criteria  1. Unable or unwilling to provide informed consent  2. Participating in another study that may affect the outcomes of this feasibility study or that does not permit co-enrolment in another study or where co-enrolment would be burdensome to the patient.  3. Contraindications to following trial treatments (e.g., following a low protein diet or co-morbidities which preclude participation in physical exercise) |
| 15 | Study type | Feasibility study for a randomised controlled trial |
| 16 | Date of first enrollment | Opened to recruitment on 15^th^ December 2022 |
| 17 | Target sample size | 381 |
| 18 | Recruitment status | Recruiting |
| 19 | Primary outcomes | 1. Eligibility rate (i.e. proportion of all on list >=65 who are frail)  2. Recruitment rates, calculated as the percentage of eligible patients recruited each month, as recorded in the recruitment logs at each site  3. Retention rates, calculated as the number of participants who complete data collection measures and/or the intervention  4. Adherence to intervention, assessed by analysis of the self-reported log on exercise and protein supplement consumption.  5. Acceptability of the trial and the intervention, evaluated through qualitative semi-structured interviews with a sample of the trial participants  6. Data completion rates, assessed by calculating data completeness for all measures  7. Data to estimate sample size required for a definitive trial |
| 20 | Key secondary outcomes | N/A |
