# Supplementary material for: Prehabilitation for frail patients undergoing total hip or knee replacement: protocol for the Joint PREP feasibility randomised controlled trial
Source: Pilot Feasibility Stud. 2023 Aug 7;9:138. doi: 10.1186/s40814-023-01363-6 (PMC10405490; doi:10.1186/s40814-023-01363-6)
Supplement: Supplementary file 2 — Additional file 2. TIDieR (Template for Intervention Description and Replication) Checklist. [file 40814_2023_1363_MOESM2_ESM.docx]

**Additional file 2: TIDieR (Template for Intervention Description and Replication) Checklist**

| **Item number** | **Item** | **Where located** |  |
| --- | --- | --- | --- |
|  |  |  | |
|  | **BRIEF NAME** |  | |
| **1.** | Provide the name or a phrase that describes the intervention. | Methods – design | |
|  | **WHY** |  | |
| **2.** | Describe any rationale, theory, or goal of the elements essential to the intervention. | Background | |
|  | **WHAT** |  | |
| **3.** | Materials: Describe any physical or informational materials used in the intervention, including those provided to participants or used in intervention delivery or in training of intervention providers. Provide information on where the materials can be accessed (e.g. online appendix, URL). | Methods – intervention: exercise  Methods – intervention: protein  Methods – intervention: intervention training | |
| **4.** | Procedures: Describe each of the procedures, activities, and/or processes used in the intervention, including any enabling or support activities. | Methods – intervention: exercise  Methods – intervention: protein  Methods – intervention: Telephone follow-up calls | |
|  | **WHO PROVIDED** |  | |
| **5.** | For each category of intervention provider (e.g. psychologist, nursing assistant), describe their expertise, background and any specific training given. | Methods – intervention: intervention training | |
|  | **HOW** |  | |
| **6.** | Describe the modes of delivery (e.g. face-to-face or by some other mechanism, such as internet or telephone) of the intervention and whether it was provided individually or in a group. | Methods – intervention: exercise  Methods – intervention: protein  Methods – intervention: Telephone follow-up calls | |
|  | **WHERE** |  | |
| **7.** | Describe the type(s) of location(s) where the intervention occurred, including any necessary infrastructure or relevant features. | Methods – intervention: exercise  Methods – intervention: protein  Methods – intervention: Telephone follow-up calls | |
|  | **WHEN and HOW MUCH** |  | |
| **8.** | Describe the number of times the intervention was delivered and over what period of time including the number of sessions, their schedule, and their duration, intensity or dose. | Methods – intervention: exercise  Methods – intervention: protein  Methods – intervention: Telephone follow-up calls | |
|  | **TAILORING** |  | |
| **9.** | If the intervention was planned to be personalised, titrated or adapted, then describe what, why, when, and how. | Methods – intervention: exercise | |
|  | **MODIFICATIONS** |  | |
| **10.^ǂ^** | If the intervention was modified during the course of the study, describe the changes (what, why, when, and how). | N/A for protocol | |
|  | **HOW WELL** |  | |
| **11.** | Planned: If intervention adherence or fidelity was assessed, describe how and by whom, and if any strategies were used to maintain or improve fidelity, describe them. | Methods – intervention: assessment of adherence | |
| **12.^ǂ^** | Actual: If intervention adherence or fidelity was assessed, describe the extent to which the intervention was delivered as planned. | N/A for protocol | |
